# Biomaterials and Tissue Engineering in Neurosurgery: Current Innovations and Future Directions

**DOI:** 10.3390/biotech14030065

**Published:** 2025-08-26

**Authors:** Jagoš Golubović, Damjan Vučurović

**Affiliations:** 1 Faculty of Medicine, University of Novi Sad, Hajduk Veljkova 3, 21000 Novi Sad, Serbia; 2 Department of Neurosurgery, University Clinical Centre of Vojvodina, 21000 Novi Sad, Serbia; 3 Department of Biotechnology, Faculty of Technology Novi Sad, University of Novi Sad, Bulevar Cara Lazara 1, 21000 Novi Sad, Serbia; dvdamjan@uns.ac.rs

**Keywords:** biomaterials, tissue engineering, neurosurgery, neural repair, cranial implants, spinal cord injury, nerve regeneration, bioprinting, nanotechnology, microfluidics

## Abstract

Neurosurgery is undergoing a significant transformation driven by advances in biomaterials and tissue engineering. These interdisciplinary innovations address challenges in repairing and regenerating neural tissues, integrating cranial and spinal implants, and improving patient outcomes. The incidence of neurological injuries such as traumatic brain injury and spinal cord injury remains high, underscoring the need for improved therapeutic strategies. This review provides a comprehensive overview of current biomaterial and tissue engineering approaches in neurosurgery, highlighting developments in neural tissue repair, cranial and spinal implants, spinal cord injury treatment, and peripheral nerve regeneration. Key challenges—such as ensuring biocompatibility, modulating the immune response, and bridging the gap between laboratory research and clinical application—are discussed. Emerging technologies including 3D bioprinting, nanotechnology (removing microfluidics), and microfluidics are examined for their potential to revolutionize neurosurgical treatments. The need for interdisciplinary collaboration among neurosurgeons, material scientists, and biologists is emphasized as critical for overcoming translational barriers and accelerating the clinical translation of these promising technologies.

## 1. Introduction

Neurosurgical practice has increasingly embraced biomaterials and tissue engineering to tackle complex neurological disorders and injuries. Advances in this field offer new solutions for issues ranging from traumatic injuries to degenerative diseases. Historically, neurosurgeons repaired skull and spinal defects using metals, bone grafts, and simple plastics; today’s biomaterials go beyond structural fixes to enable tissue regeneration. For example, in 2024, there were an estimated 2.8 million cases of traumatic brain injury globally leading to substantial disability and healthcare burdens [1]. Traditional neurosurgical approaches often struggle to fully restore function in these cases, which motivates the exploration of regenerative strategies. Biomaterials provide physical scaffolding and bioactive cues for tissue repair, while tissue engineering integrates cells and biofactors to recreate neural tissues in vitro and enhance in vivo healing [2]. Recent breakthroughs have demonstrated improved outcomes in animal models of neural injury using engineered scaffolds and stem cell therapies. Despite this progress, significant challenges remain in translating these therapies to patients. Issues such as immune rejection, the limited regenerative capacity of the central nervous system tissue, and the complex microenvironment of the brain and spinal cord must be addressed [3]. This review covers the current state of biomaterials and tissue engineering in neurosurgery, focusing on applications in three key areas: (I) brain tissue repair via engineered scaffolds and hydrogels, (II) spinal cord injury and spinal stabilization using implants and regenerative constructs, and (III) peripheral nerve repair via guidance conduits and hybrid scaffolds. Furthermore, we discuss how emerging technologies like 3D bioprinting, nanotechnology, and microfluidic organ-on-chip models are poised to drive future innovations. The goal is to provide neurosurgeons and researchers with a detailed understanding of these advances and to outline future directions for integrating biomaterials into neurosurgical care.

## 2. Materials and Methods

This literature review was conducted by surveying research articles and clinical studies from the past decade (2015–2025) focusing on biomaterials and tissue engineering applications in neurosurgery. Database searches were performed in PubMed, Web of Science, and Google Scholar using keywords such as “neural tissue engineering”, “biomaterial scaffold neurosurgery”, “spinal cord injury regeneration”, and “nerve conduit”. Relevant articles were selected for inclusion based on their contribution to the topics of neural repair, cranial/spinal implants, spinal cord injury, nerve regeneration, and emerging technologies (bioprinting, nanotechnology, microfluidics). Both in vitro experimental studies, in vivo animal studies, and any available clinical trial results were reviewed. No human subjects or new experimental data were generated for this review; thus, no institutional ethical approval was required. The findings are organized into thematic sections corresponding to major application areas.

Study Quality Assessment: Study quality was assessed using standardized criteria (e.g., risk-of-bias tools) for the included papers. We noted limitations such as heterogeneous study designs (ranging from cell culture to animal models), lack of randomized controlled trials in preclinical research, and publication bias toward positive outcomes. These factors limit the ability to directly compare results across studies. Additionally, this narrative review did not perform a quantitative meta-analysis, which is a limitation; quantitative effect sizes are not provided. We acknowledge these methodological constraints and have interpreted results in that context.

## 3. Results

**Figure 1:**
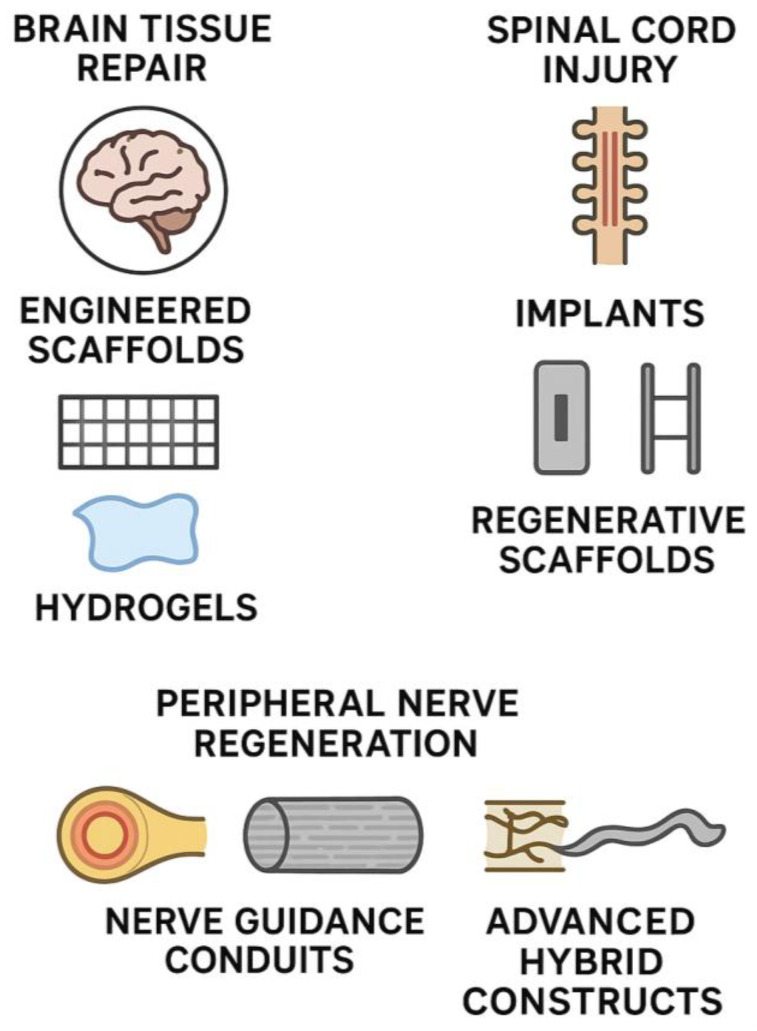
Schematic of biomaterials and tissue engineering strategies in neurosurgery. Brain tissue repair employs engineered scaffolds and injectable hydrogels to mimic extracellular matrix properties and promote regeneration. Spinal cord injury management combines structural implants for stabilization with regenerative scaffolds seeded with stem cells or growth factors. Peripheral nerve regeneration relies on nerve guidance conduits and advanced hybrid constructs (e.g., nanofiber-coated or bioactive conduits) to restore axonal continuity and function.

Section Overview: The following section is organized by neurosurgical application area. First, we address biomaterial strategies for brain tissue repair (e.g., after traumatic brain injury or stroke, etc.). Next, we examine approaches for spinal cord injury and spine stabilization (including spinal column implants and tissue-engineered constructs for cord repair). Finally, we cover biomaterial approaches to peripheral nerve repair. In each subsection, we highlight the clinical relevance, key materials and methods, and examples of outcomes in relevant models (Figure 1).

### 3.1. Brain Tissue Repair

Conventional medicine often lacks effective treatments for neural injuries, typically focusing on symptom management. Neural tissue repair poses unique challenges due to the limited regenerative capacity of the central nervous system and the intricate architecture of neural networks. Engineered scaffolds have been developed to provide a structural and biochemical framework that encourages neural regeneration. The design of these scaffolds must balance mechanical support with a permissive environment for cell growth and connectivity. Materials selection is critical: natural biomaterials (e.g., collagen, fibrin, chitosan) offer excellent biocompatibility and contain intrinsic cell-binding motifs, whereas synthetic polymers [such as poly(lactic acid) (PLA), poly(glycolic acid) (PGA), and polycaprolactone (PCL)] provide tunable mechanical properties and degradation rates [4]. Recent scaffold designs emphasize biomimicry of the native extracellular matrix (ECM) of neural tissue. For instance, porous collagen or hyaluronic acid-based scaffolds can closely resemble neural ECM in composition and stiffness, promoting neuron and glial cell attachment and growth. In addition, composite approaches combine natural and synthetic components to harness the advantages of each; an example is a collagen—PGA composite scaffold that offers cell-adhesive sites alongside long-term structural stability [5]. In neurosurgical practice, such scaffolds could be implanted into a brain resection cavity (for example, after tumor removal) to fill the void and deliver bioactive signals to the surrounding tissue.

#### 3.1.1. Hydrogels

**Figure 2:**
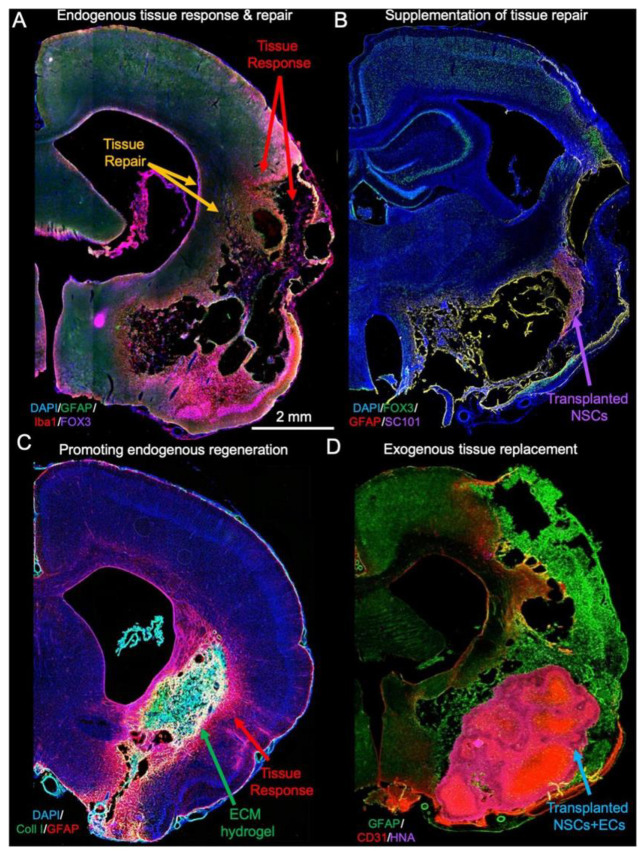
Integration of an ECM-based hydrogel scaffold into a rodent brain lesion. (**A**) endogenous tissue response and repair, (**B**) Supplementation of tissue repair, (**C**) Promoting endogenous regeneration, (**D**) Exogenous tissue replacement. Histological images from Modo et al. [9] demonstrate persistence of the scaffold up to 90 days after implantation, with extensive host cell infiltration, neovascularization, and reduced glial scarring. These findings highlight the capacity of engineered bioscaffolds to create a permissive environment for tissue regeneration. (Reproduced under CC BY 4.0).

Hydrogels are a class of injectable biomaterials that closely match the soft mechanics of brain tissue, making them attractive for filling irregular cavities. Common hydrogel materials include agarose, alginate, PEG, and hyaluronic acid, often chemically crosslinked in situ after injection. These hydrogels can act as depots for the controlled release of therapeutic molecules. For example, in a rodent stroke model, a PEG-based hydrogel loaded with neurotrophic factors like BDNF or NGF was injected into the lesion cavity; it gradually released these proteins over several weeks, enhancing neuronal survival and axon extension compared to the untreated lesions. Similarly, hydrogels containing anti-inflammatory drugs or antibodies (e.g., anti-Nogo-A) have been investigated to mitigate the inhibitory scar environment and promote regeneration after CNS injury [6,7]. One advantage of injectable hydrogels is minimal invasiveness: they can be delivered via catheter or syringe into the brain or spinal lesion during surgery, conforming to the cavity shape. However, challenges include controlling gelation time (so it does not set before distribution) and preventing rapid hydrogel contraction or degradation (as observed in un-crosslinked ECM gels) [8]. As illustrated in Figure 2 (reproduced under CC BY 4.0 from Modo et al., 2019), ECM-based hydrogel scaffolds achieved long-term integration with the host brain tissue—supporting cellular infiltration and neovascularization while limiting glial scarring—reinforcing the translational potential of these biomaterials for neurosurgical repair [9].

#### 3.1.2. Nanomaterials and Fiber Scaffolds

The incorporation of nanomaterials imparts new functions to neural scaffolds. Conductive nanomaterials (carbon nanotubes, graphene, conductive polymers) can be embedded to provide electrical conductivity, which is beneficial for excitable neural networks. For example, graphene- or PEDOT-coated scaffolds have supported enhanced neurite outgrowth in culture by facilitating electrical signaling. Electrospun nanofibers offer another strategy: aligned polymer or ECM fibers guide the neurite extension along defined tracks. In one design, aligned electrospun gelatin fibers coated with laminin supported Schwann cell alignment and robust axon growth in vitro. These fibers can mimic the linear structure of axon bundles. Nanofibrous scaffolds, however, may have limited cell infiltration unless engineered with larger pores or 3D knitting [10,11,12].

#### 3.1.3. Biocompatibility and Immune Modulation

The brain’s immune environment is delicate, so any implant must minimize adverse reactions. Biomaterials can elicit inflammation and glial scarring if not carefully designed. Strategies to improve biocompatibility include using decellularized neural ECM (dECM) scaffolds, which preserve native proteins and glycosaminoglycans. Neural dECM, processed into a hydrogel or coating, has been shown to promote neuronal and stem cell adhesion and differentiation while reducing foreign-body response [13,14]. Surface modifications also help; for example, coating a scaffold with polyethylene glycol can reduce protein adsorption and mitigate initial inflammatory reactions. In experimental models, modulating macrophage polarization has improved outcomes; scaffolds pre-loaded with anti-inflammatory cytokines (e.g., IL-10) or with peptides that encourage M2 (pro-regenerative) macrophage phenotypes have reduced chronic inflammation [15,16]. In practice, neurosurgeons must consider that an implanted hydrogel or scaffold will interact with astrocytes and microglia; thus, many designs now include anti-inflammatory features to prevent scar formation (e.g., slow-release dexamethasone from the matrix).

### 3.2. Spinal Cord Injury and Spinal Implants

The neurosurgical management of spine issues involves both mechanical stabilization and functional repair. For spinal stabilization, implants (cages, rods, plates) are routinely used during fusion surgeries or fracture fixation. Historically, titanium hardware has been standard for spinal fusion devices. However, newer polymers address some limitations of metal. Polyether ether ketone (PEEK) is now widely used for interbody cages and rods: its elastic modulus (~3 GPa) is closer to bone than titanium, reducing stress shielding. PEEK is radiolucent, which allows clearer post-op imaging; however, it is bioinert and does not integrate with bone unless treated. Therefore, surface treatments such as hydroxyapatite coating or introducing porosity on PEEK devices have been applied to enhance bone ongrowth. Clinically, PEEK cages are used in lumbar fusion to restore disc height while enabling a more natural load transfer [17,18]. Advanced approaches include 3D-printed spinal implants with patient-specific geometry, using materials (e.g., titanium or PEEK) designed to match the patient’s vertebral anatomy [19,20]. These developments underscore how biomaterials integrate with standard spine surgery practice to improve outcomes.

#### 3.2.1. Tissue Engineering for Spinal Cord Repair

Parallel to spinal stabilization is the challenge of repairing the spinal cord itself after injury. Tissue engineering strategies for SCI aim to bridge the gap in the cord and promote axon regrowth. Scaffold design: Experiments have shown that multi-channel collagen or polymer scaffolds, seeded with cells, can guide axons. For example, a multi-channel collagen scaffold seeded with neural stem cells (NSCs) was implanted into a transected rat spinal cord; it provided aligned paths for axons to grow across the lesion, yielding better motor recovery than the controls. Scaffolds can include channels or oriented fibers to maintain axon trajectories and prevent them from wandering into scar tissue. These constructs are typically pre-seeded with cells (e.g., NSCs, mesenchymal stem cells, or Schwann cells) before implantation [6,11]. In rodent models, NSCs delivered within fibrin or collagen scaffolds have differentiated into neurons and glia that integrate with the host tissue, contributing to partial functional recovery. The scaffold protects the cells and retains growth factors, boosting survival relative to cell injection alone. Stem cell sources: NSCs and neural progenitors can replace lost neurons and remyelinate axons. Induced pluripotent stem cell (iPSC)-derived neural progenitors offer patient-specific therapy without ethical issues, though tumor risk must be managed. MSCs secrete neurotrophic factors and modulate inflammation and can be included to enhance the local environment [21,22]. In practice, during surgical decompression for SCI, a neurosurgeon might implant such a scaffold at the injury site.

#### 3.2.2. Bioactive Molecules and Controlled Delivery in SCI

The inhibitory environment of a spinal injury often requires additional biochemical cues. Biomaterial vehicles are used to locally deliver factors like neurotrophins or enzymes. For instance, chondroitinase ABC can degrade the glial scar’s CSPGs; when slowly released from a polymer microsphere in a scaffold, it reduces inhibitory barriers and permits axon extension. In one study, combining chondroitinase release with sustained BDNF release from the same scaffold synergistically enhanced axon growth through a rat spinal lesion. Other growth factors (NT-3, GDNF) can be released from embedded microspheres or heparin-based gels over several weeks, supporting specific neuron populations [23,24]. Controlled release addresses the fact that systemic delivery often fails to reach the cord at a sufficient dose. For example, NT-3 in a gradient along a scaffold promoted corticospinal tract regrowth in animal models. In the surgical setting, these biomaterial-factored constructs could be applied to the injured cord to provide a timed-release therapeutic environment [11,25]. There are early-phase clinical trials testing scaffold-assisted cell or factor therapies in subacute human SCI, which will shed light on safety and feasibility [26,27].

### 3.3. Peripheral Nerve Regeneration

Peripheral nerve injuries (e.g., brachial plexus or extremity trauma) benefit from regenerative approaches because these nerves have some innate ability to heal. The gold standard is autologous nerve grafting, but this requires sacrificing a donor nerve (with morbidity). Engineered nerve guidance conduits (NGCs) have been developed to provide an alternative. An NGC is a tubular scaffold that connects the two ends of a severed nerve, guiding axons across the gap. Early conduits were simple inert tubes; modern ones are biodegradable and bioactive. Common materials include collagen, gelatin, chitosan, PCL, and PLGA, chosen for their biocompatibility and resorbability. For example, FDA-approved collagen tube conduits are used for short gaps (<20 mm), where they support axon growth along the conduit wall. Chitosan conduits (from chitin) show antibacterial properties and comparable performance to autografts in animal studies for short gaps [28,29,30].

Important design features include the internal microarchitecture. Many modern NGCs incorporate longitudinal fibers or microchannels: these aligned inner structures further direct axons and improve support. For instance, adding multiple longitudinal channels inside a conduit improved nerve regeneration across a 10 mm gap in a rat sciatic model compared to a hollow tube. Conduits may also be impregnated with Schwann cells or growth factor gradients (NGF, GDNF) to boost regeneration. In practice, a neurosurgeon repairing a nerve would suture the conduit ends to the nerve stumps during surgery, placing this engineered graft instead of an autograft. Recent advances include conduits fabricated by 3D printing to patient-specific sizes and geometries, and nanofiber-coated conduits that mimic the extracellular matrix cues for axons. Importantly, preclinical studies have demonstrated functional recovery (muscle reinnervation and improved limb function) using advanced conduits, although complete recovery in long gaps (>3 cm) remains a challenge [1,31,32].

The results summarized below (Table 1) demonstrate significant progress in developing biomaterial-based interventions for neurosurgical applications. In the following section, we discuss the broader implications of these findings, the remaining challenges, and future directions for integrating emerging technologies into clinical practice.

## 4. Discussion

The convergence of biomaterial science and neurosurgery is generating novel therapeutic possibilities for conditions previously deemed irreversible. Engineered scaffolds and smart implants can address key issues by replacing lost neural cells (with cell-seeded matrices), guiding axonal regrowth across injuries, and reconstructing bony defects (with patient-specific implants) [41]. However, translating these experimental successes into routine treatments requires surmounting several challenges.

One major challenge is ensuring the long-term safety and stability of the implanted biomaterials. While many materials show excellent short-term biocompatibility, their behavior over years in the human CNS is less understood. Chronic immune responses or byproducts of degradation could potentially cause delayed inflammation or toxicity. For example, early conductive scaffolds with carbon nanotubes enhanced neurite outgrowth, but persistent nanotubes raise concerns about long-term neuroinflammation. Therefore, rigorous long-term biocompatibility studies are needed, and the development of materials that degrade into inert byproducts remains a priority [2,42].

From a manufacturing perspective, the consistency and scalability of advanced biomaterials must be achieved. Techniques like 3D printing and decellularization enable complex grafts, but they must be standardized under good manufacturing practices to produce reproducible scaffolds. Cost is another issue: personalized or cell-laden implants can be expensive; innovations (e.g., off-the-shelf allogeneic constructs or automated cell culture) will be necessary to make these therapies accessible. Regulatory pathways will demand a demonstration of safety and efficacy for each new device or scaffold, meaning only the most robust technologies will reach trials [43,44].

Pros and Cons of Major Approaches: Each biomaterial strategy has trade-offs. *Hydrogel scaffolds*, for instance, can be delivered minimally invasively and can carry cells/drugs, but they may contract or degrade too rapidly in vivo, potentially losing their structure before the tissue regenerates [45]. *Nanofiber mats* closely mimic ECM architecture and orient axons, yet often require enhancements (e.g., porosity or inclusion of supportive gels) to allow cell infiltration beyond the surface [46]. *Rigid implants* (PEEK, titanium) restore anatomy robustly but lack biological activity; they must be surface-modified or combined with osteoconductive materials to achieve integration. For example, a titanium skull plate provides immediate protection, but, without a hydroxyapatite coating, may not bond to the bone, risking long-term loosening [47]. *Nerve conduits* spare the patient a donor graft and successfully repair short nerve gaps, but functional recovery in longer gaps (>30 mm) is incomplete and remains a limitation. In each case, the advantages (e.g., precise anatomic fit, delivery of growth factors) must be weighed against the limitations (e.g., limited cell migration, need for multiple surgeries, or variable degradation rates) [39].

Despite these hurdles, several emerging technologies stand out as promising avenues to propel the field: bioprinting and advanced manufacturing, nanotechnology-enabled delivery, and hybrid decellularized tissue constructs [48]. Three-dimensional bioprinting is evolving toward creating living neural tissues with vascular channels; researchers have printed neural-cell-laden constructs with predefined architecture. In the future, bioprinted neural grafts could be implanted into stroke cavities to replace lost brain tissue. Key challenges—the survival of thick implants and integration with host circuits—are being addressed by perfusion channels and patient-specific cell sourcing [49]. Nanotechnology offers targeted delivery: for instance, nanoparticles engineered to cross the blood–brain barrier could deliver neuroprotective agents systemically to injury sites. Embedded nanosensors in implants could report the local conditions (e.g., pH or oxygenation) to clinicians in real time [50]. Decellularized and hybrid constructs leverage nature’s design: whole-organ decellularization (e.g., an acellular spinal cord scaffold) seeded with patient cells is an area of exploration. Combining synthetic matrices (for mechanical strength) with dECM (for signaling) appears particularly attractive; early studies show that even the partial regeneration of spinal or nerve segments can reconnect severed ends [51,52].

## 5. Conclusions

In conclusion, recent advances in biomaterials and tissue engineering hold great promise to improve outcomes in neurosurgery. The most impactful findings include demonstrations that engineered scaffolds can support neural regeneration in preclinical models (e.g., nerve fibers growing through collagen conduits), and that advanced implants (PEEK, 3D-printed titanium) can reconstruct the anatomy with fewer complications. Notably, studies combining multiple elements—such as a cell-loaded scaffold that releases growth factors—consistently yield better regeneration than single-factor treatments. Among the approaches reviewed, nerve guidance conduits for peripheral nerve repair and bioactive cranial implants are already the closest to clinical use, given the existing FDA-approved conduits and growing trials of custom cranioplasty plates. For CNS repair, the most promising near-term strategy may be collagen or hyaluronic acid hydrogels delivering neural stem cells, which are entering early clinical testing. We anticipate that, in the next few years, one or two of these biomaterial-based therapies (e.g., a scaffold for spinal repair or an engineered nerve graft) could move into broader clinical application. Continued interdisciplinary research and translation efforts are essential to realize the full potential of these technologies in neurosurgical practice.

## Figures and Tables

**Table 1:** Summary of biomaterials and tissue engineering in neurosurgery.

Category	Approach/Subtype	Key Materials/Strategies	Clinical/Experimental Notes	Reference
Brain Tissue Repair	Engineered scaffolds	Natural biomaterials (collagen, fibrin, chitosan); synthetic polymers (PLA, PGA, PCL); composites (e.g., collagen–PGA)	Provide mechanical support + bioactive environment; used to fill resection cavities; mimic ECM to promote growth.	Smith et al., 2025. [33]
	Hydrogels	Agarose, alginate, PEG, hyaluronic acid hydrogels; in situ crosslinking; drug/antibody-loaded (BDNF, NGF, anti-Nogo-A)	Injectable; conform to cavity shape; depot for controlled release; shown effective in rodent stroke models.	Xu et al., 2023. [34]
	Nanomaterials and fiber scaffolds	Conductive scaffolds (graphene, PEDOT, CNTs); electrospun gelatin/laminin fibers	Conductivity enhances neurite outgrowth; aligned fibers guide axons; limited infiltration unless modified.	Licciardello et al., 2024. [35]
	Biocompatibility/immune modulation	Decellularized neural ECM; PEGylated surfaces; scaffolds with IL-10 or M2-polarizing peptides; slow-release dexamethasone	Reduces glial scarring and chronic inflammation; improves cell adhesion and integration in CNS tissue.	Kim et al., 2021. [36]
Spinal Cord Injury and Implants	Spinal stabilization implants	Titanium and PEEK cages/rods; hydroxyapatite coatings; 3D-printed patient-specific spinal implants	Mechanical stability in trauma/degeneration; PEEK reduces stress shielding; imaging compatibility.	Cheers et al., 2024. [37]
	Tissue-engineered cord repair	Multi-channel collagen/polymer scaffolds seeded with NSCs, MSCs, Schwann cells	Guides axon regrowth in transected cord models; cells differentiate and integrate; partial motor recovery achieved.	Da Silva et al., 2023. [38]
	Bioactive molecule delivery	Scaffolds/microspheres releasing BDNF, NT-3, GDNF, chondroitinase ABC	Sustains local factor delivery; degrades scar CSPGs; promotes remyelination and axon extension.	Mungenast et al., 2023. [14]
Peripheral Nerve Regeneration	Nerve guidance conduits (NGCs)	Biodegradable tubes (collagen, gelatin, chitosan, PCL, PLGA); FDA-approved collagen conduits; microchannel or fiber-lined conduits	Bridge short gaps (<20 mm); support axon guidance; alternatives to autografts; some FDA-approved devices.	Yu et al., 2023. [39]
	Advanced conduits	The 3D-printed customized conduits; nanofiber-coated or electroconductive hybrids; Schwann cell- or NGF/GDNF-loaded conduits	Improve regeneration across longer gaps; mimic ECM cues; show muscle reinnervation in animal models.	Liu et al., 2024. [40]

## Data Availability

No new data were created.

## Abbreviations

The following abbreviations are used in this manuscript:

TBI	Traumatic Brain Injury
SCI	Spinal Cord Injury
CNS	Central Nervous System
ECM	Extracellular Matrix
PLA	Poly(lactic acid)
PGA	Poly(glycolic acid)
PCL	Polycaprolactone
PEG	Polyethylene Glycol
HA	Hyaluronic Acid
BDNF	Brain-Derived Neurotrophic Factor
NGF	Nerve Growth Factor
CNTs	Carbon Nanotubes
PEDOT	Poly(3,4-ethylenedioxythiophene)
dECM	Decellularized Extracellular Matrix
IL-10	Interleukin-10
PEEK	Polyether Ether Ketone
NSCs	Neural Stem Cells
MSCs	Mesenchymal Stem Cells
iPSC	Induced Pluripotent Stem Cell
NT-3	Neurotrophin-3
GDNF	Glial Cell Line-Derived Neurotrophic Factor
CSPGs	Chondroitin Sulfate Proteoglycans
NGCs	Nerve Guidance Conduits
PLGA	Poly(lactic-co-glycolic acid)
FDA	Food and Drug Administration
